# What is the best adjustment of appendicular lean mass for predicting mortality or disability among Japanese community dwellers?

**DOI:** 10.1186/s12877-017-0699-6

**Published:** 2018-01-05

**Authors:** Rei Otsuka, Yasumoto Matsui, Chikako Tange, Yukiko Nishita, Makiko Tomida, Fujiko Ando, Hiroshi Shimokata, Hidenori Arai

**Affiliations:** 1 Section of NILS-LSA (National Institute for Longevity Sciences–Longitudinal Study of Aging), Center for Gerontology and Social Science, National Center for Geriatrics and Gerontology, 7-430 Morioka-cho, Obu, Aichi 474-8511 Japan; 2 Department of Orthopedics, National Center for Geriatrics and Gerontology, 7-430 Morioka-cho, Obu, Aichi 474-8511 Japan; 3 Faculty of Health and Medical Sciences, Aichi Shukutoku University, 2-9 Katahira, Nagakute, Aichi 480-1197 Japan; 4 Graduate School of Nutritional Sciences, Nagoya University of Arts and Sciences, 57 Takeyanoyama, Iwasaki-cho, Nisshin, Aichi 470-0196 Japan; 5 Hospital, National Center for Geriatrics and Gerontology, 7-430 Morioka-cho, Obu, Aichi 474-8511 Japan

**Keywords:** Japanese, Skeletal muscle mass, Sarcopenia, Criteria, Mortality, Disability

## Abstract

**Background:**

Age-related declines in skeletal muscle mass and strength, representing “sarcopenia,” are a growing concern in aging societies. However, the prevalence of low muscle mass based on the height^2^-adjustment has been shown to be extremely low, and a more appropriate definition of low muscle mass is needed, particularly for Asian women. The aim of this study was to explore the most appropriate adjustment of appendicular lean mass (ALM) for predicting mortality or disability risk using ALM or any of 5 adjustments of ALM among community-dwelling Japanese.

**Methods:**

Subjects comprised 1026 men and 952 women between 40 and 79 years old at baseline (1997–2000) who participated in the National Institute for Longevity Sciences - Longitudinal Study of Aging, Japan. ALM (kg) and 5 adjusted indices of ALM (ALM/leg length, ALM/height, ALM/height^2^, ALM/weight, and ALM/body mass index [BMI]) were assessed at baseline. Disability was defined by long-term care insurance certification based on responses to a survey mailed in 2013, and death records were obtained as vital statistics until December 2014. Crude and adjusted Cox proportional hazard models were used to estimate hazard ratios for mortality or disability by sex-stratified quintiles of each ALM index (ALM and adjusted ALM) or sarcopenia-related indices. The area under the curve (AUC) was calculated with the multivariate-adjusted logistic regression model. Additionally, mixed-effects analyses were used to clarify the age-related ALM indices decline over 12 years (*n* = 1838).

**Results:**

Crude Cox proportional hazard models and multivariate-adjusted logistic model (AUC) indicated that higher ALM and ALM/BMI in women, and higher ALM, ALM/leg length, ALM/height, and ALM/BMI in men were associated with lower risks for mortality or disability than ALM/height^2^. The mixed effect model indicated all ALM indices in men, and ALM, ALM/leg length, and ALM/height in women could better predict age-related lean muscle mass decline.

**Conclusions:**

Unadjusted ALM in women, and ALM/leg length, ALM/height, ALM/BMI, and ALM in men may be more appropriate for predicting future mortality or disability than ALM/height^2^. Considering the age-related muscle mass decline, unadjusted ALM would be the first variable to assess, regardless of sex, in this Japanese cohort study.

**Electronic supplementary material:**

The online version of this article (10.1186/s12877-017-0699-6) contains supplementary material, which is available to authorized users.

## Background

Age-related declines in skeletal muscle mass and strength, referred to as “sarcopenia” [1–3], are a growing concern in Asian countries as the population ages [4], because sarcopenia is associated with adverse health outcomes in older adults [5]. In 2014, the Asian Working Group for Sarcopenia (AWGS) announced the development of a diagnostic algorithm for sarcopenia for Asians [6]. The AWGS recommended using height^2^-adjusted skeletal muscle mass index (SMI) values rather than weight-adjusted SMI values [2, 7]. However, the prevalence of low muscle mass based on the height^2^-adjustment among Asian women was shown to be extremely low [8], and height^2^-adjusted muscle mass was not shown to decrease with age [9, 10].

The Foundation for the National Institutes of Health (FNIH) Sarcopenia Project proposed “appendicular lean mass (ALM) adjusted body mass index (BMI)” as cut-off points for low lean mass in men and women in 2014 [11]. The sarcopenia criteria using these definitions by FNIH better predicted mortality among Korean men, and there was no positive association in women [12]. However, using the lowest quintile in that study showed better predictive value to estimate mortality in women than using ALM/height^2^. An alternative and more appropriate definition of low muscle mass is still needed [13], particularly for Asian women.

The research question in this study was “what is the best adjustment of appendicular lean mass for predicting mortality or disability among Japanese community dwellers?” To explore the most appropriate skeletal muscle mass adjustment in Asians, we measured total ALM and used 5 different adjustments—ALM/leg length, ALM/height, ALM/height^2^, ALM/weight, and ALM/body mass index (BMI)—to examine the associations of ALM and each adjustment with mortality or disability among community-dwelling Japanese individuals. Four of 5 of these skeletal muscle mass adjustments were chosen according to previous studies [14–16], and we added ALM/leg length adjustment as a fifth variable. We hypothesized leg length for ALM adjustment would better predict age-related ALM decline than using height adjustments because we considered adjustments using height^2^ might result in over-adjustment for the elderly, as upper body height, especially in women, tends to shorten with age.

## Methods

### Study cohort

Data were collected as part of the National Institute for Longevity Sciences–Longitudinal Study of Aging (NILS-LSA). In this project, the normal aging process has been assessed over time using detailed questionnaires, medical check-ups, anthropometric measurements, physical fitness tests, and nutritional examinations. Participants in the NILS-LSA included randomly selected age- and sex-stratified individuals from the non-institutionalized residents in the institute neighbourhood areas of Obu City and Higashiura Town in Aichi Prefecture in Japan. The first wave of the NILS-LSA was conducted from 1997 to 2000 and included 2267 participants (1139 men, 1128 women; age range, 40–79 years). Details of the NILS-LSA study have been reported elsewhere [17]. Subjects were followed-up every 2 years from the second to seventh wave (2000–2012).

### Follow-up survey and vital statistics records

In July 2013, a self-administered questionnaire was mailed to participants to assess health status, including a “requirement for long-term care” under the new long-term care insurance system that started in Japan in 2000 [18, 19]. In addition, we obtained death records for all participants and obtained information from local government regarding which participants had moved to other areas. To clarify causes of death, we used National Vital Statistics records that were available until the end of December 2014. Mortality or disability was defined according to the National Vital Statistics records, or self-reported long-term care insurance certification, respectively. The main outcome in this study was “composite outcome for mortality or disability” as we combined these outcomes to increase the statistical power (to increase the number of cases).

### Study subjects

**Fig. 1:**
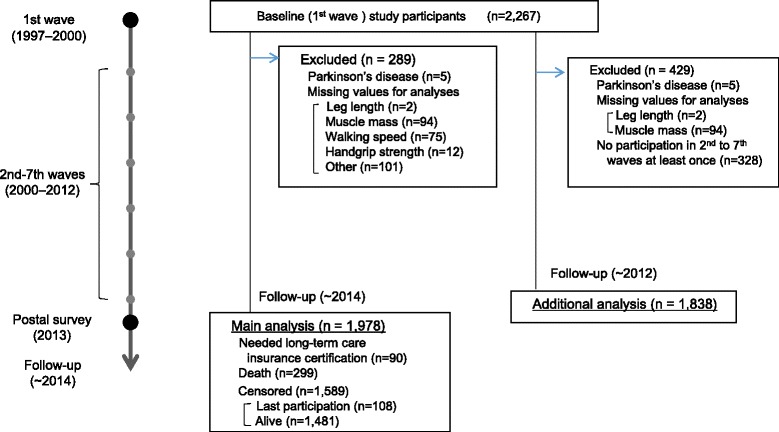
Flow chart of study subjects in the National Institute for Longevity Sciences–Longitudinal Study of Aging

Among the 2267 participants who participated in the first wave, we excluded 289 patients with a history of Parkinson’s disease (*n* = 5) or for whom data were missing (*n* = 284) (Fig. 1). Of the 1978 participants still being followed as of 2014, 389 men and women were categorized as having died (*n* = 299) or as needing long-term care insurance certification (*n* = 90). Of the 1589 men and women categorized as “censored,” 1481 were confirmed as alive according to information from the local government, and 108 had moved away from the local area or dropped out.

The study protocol was reviewed and approved by the Committee of Ethics of Human Research at the National Center for Geriatrics and Gerontology. Written informed consent was obtained from all subjects during the first to the seventh wave of the NILS-LSA. In the survey mailed in 2013, we explained that returning the self-administered questionnaire represented informed consent. Death data were obtained by means of secondary usage of demographic statistics by predetermined procedures. We used anonymous death data that had already been collected by the Ministry of Health, Labour and Welfare in Japan and do not require consent of individuals.

#### Assessment of muscle mass

ALM (kg), which represents the appendicular fat-free mass minus the bone mineral content [10], was assessed using a QDR-4500 dual-energy X-ray absorptiometry (DXA) system (Hologic, Bedford, MA).

We calculated 5 indices using ALM (kg): ALM divided by leg length (kg/m); ALM divided by height (kg/m); ALM divided by height squared (kg/m^2^); ALM divided by weight and multiplied by 100 (kg/kg*100); and ALM divided by BMI and multiplied by 10 (kg/kg/m^2^*10). BMI was calculated as weight divided by height squared.

### Other measurements

History of stroke, hypertension, hyperlipidemia, heart disease, and diabetes (past and current), current smoking status (yes/no), education (≤9 or ≥10 years of school), and annual family income (<5,500,000 or ≥5,500,000 yen per year) were collected using self-reported questionnaires, and medical doctors or trained staff confirmed the information [20]. Sarcopenia was defined according to AWGS [6] criteria using grip strength, gait speed, and muscle mass. All measurements were assessed in the first-wave survey of the NILS-LSA.

### Statistical analysis

Sex differences in baseline characteristics of participants were analysed using the t test or χ^2^ test. The difference in the prevalence of mortality or disability by sex-stratified quintiles of each ALM index (ALM and adjusted ALMs) or sarcopenia-related indices was analysed using the χ^2^ test. Crude and adjusted Cox proportional hazard models were used to estimate hazard ratios (HRs) and 95% confidence intervals (CIs) for mortality or disability by sex-stratified quintiles of each ALM index (ALM and adjusted ALMs) or sarcopenia-related indices. For Cox models, follow-up time (years) was calculated by the duration (days) that had elapsed since the day on which each participant entered the first wave of the NILS-LSA. The last day of follow-up for each participant was used for analysis, as either the date of death, the earliest day of needing long-term care (event group), the latest day of last participation in the NILS-LSA, or December 2014, whichever came first (censored group). When the participants were missing, that is, they moved away from the local area or dropped out (*n* = 108), we considered the last participation day of the NILS-LSA as the last day of follow-up.

Variables considered for adjustment were age, smoking status, education, family income, history of stroke, hypertension, heart disease, hyperlipidemia, and diabetes mellitus (multivariate-adjusted). In sub-analyses, we calculated receiver-operating-characteristic (ROC) curves on disability or mortality according to the sex-stratified quintiles of each ALM index (ALM and adjusted ALMs). The area under the ROC curve (AUC) was calculated with the multivariate-adjusted logistic regression model.

Probability levels of <.05 and <.10 were considered significant and marginally significant, respectively. All statistical analyses were conducted using Statistical Analysis System software version 9.3 (SAS Institute, Cary, NC).

### Additional analyses

To clarify age-related changes in each ALM index (ALM alone and ALM with each of the 5 adjustments), we attempted to describe trends in changes to these indices over 12 years according to age in the first wave of the study of the NILS-LSA.

Among the 2267 participants (age range, 40–79 years) who participated in the first wave, we excluded patients with a history of Parkinson’s disease (*n* = 5) or for whom data were missing (*n* = 96) (Fig. 1). We selected subjects who also participated in more than one study wave from the second to the seventh wave, because variables could be followed-up at least once from the first wave. A total of 1838 subjects (951 men, 887 women) who were in the first wave were available for analysis. Each wave was conducted every 2 years. Mean (SD) interval and participation times between the first and last waves of participation for each participant were 5.5 (4.1) years and 3.7 (2.0) times, respectively.

For repeated-measures analyses of each ALM index, a mixed-effects model was used. To estimate fixed effects on each ALM index by follow-up time, both age at baseline and the interaction of follow-up time × age were substituted into the model. To clarify the impact of habitual lifestyles on each ALM index, we additionally adjusted the mixed effect model using lifestyle-related factors including smoking status, alcohol intake, total physical activity, and energy intake.

## Results

Baseline characteristics of study participants are shown in Table 1. Mean age was 58.8 years in men and 58.5 years in women. Mean BMI was 23.0 in men and 22.8 in women. Significantly more men had diabetes, and significantly more women had dyslipidemia. Mean ALM index (ALM and adjusted ALMs) were significantly higher in men than in women.

**Table 1:** Baseline characteristics of study participants

	Men	Women	
Characteristic	(*n* = 1026)	(*n* = 952)	*P* ^*^
Age, years, mean (SD)	58.8 (10.9)	58.5 (10.8)	.588
Weight, kg, mean (SD)	62.4 (9.2)	52.6 (8.0)	<.001
Height, cm, mean (SD)	164.7 (6.4)	151.7 (5.9)	<.001
Leg length, cm, mean (SD)	80.3 (4.1)	74.2 (3.6)	<.001
BMI, kg/m^2^, mean (SD)	23.0 (2.8)	22.8 (3.1)	.455
ALM, kg, mean (SD)	20.1 (2.8)	14.1 (2.0)	<.001
ALM/leg length, kg/m, mean (SD)	25.0 (3.1)	18.9 (18.8)	<.001
ALM/height, kg/m, mean (SD)	12.2 (1.4)	9.3 (1.1)	<.001
ALM/(height)^2^, kg/m^2^, mean (SD)	7.3 (5.0)	6.1 (4.2)	<.001
ALM/weight, kg/kg × 100, mean (SD)	32.4 (2.3)	26.7 (2.3)	<.001
ALM/BMI, kg/kg/m^2^ × 10, mean (SD)	8.8 (1.0)	6.2 (0.8)	<.001
Current smoker, n (%)	388 (37.8)	69 (7.2)	<.001
Education, ≤ 9 years, *n* (%)	297 (28.9)	342 (35.9)	<.001
Family income, < 5,500,000 yen/year, *n* (%)	395 (38.5)	429 (45.1)	.003
Stroke, *n* (%)	34 (3.3)	13 (1.4)	.005
Hypertension, *n* (%)	236 (23.0)	241 (25.3)	.230
Heart disease, *n* (%)	121 (11.8)	97 (10.2)	.255
Hyperlipidemia, *n* (%)	135 (13.2)	187 (19.6)	<.001
Diabetes, *n* (%)	102 (9.9)	45 (4.7)	<.001

*SD* standard deviation, *BMI* body mass index, *ALM* appendicular lean mass

^*^The *t*-test was used for continuous variables, and the χ^2^ test was used for categorical variables

Tables 2 and 3 show the associations between baseline ALM indices and composite outcomes of mortality or disability in men and women, respectively. Before the Cox proportional hazard models, we tested the difference of the prevalence of mortality or disability by sex-stratified quintiles of each ALM index (ALM and adjusted ALMs) or sarcopenia-related indices. In men, the prevalence of mortality or disability was different according to the quintiles of all ALM indexes. In women, the prevalence was statistically different in ALM, ALM/leg length, and ALM/BMI only.

**Table 2:** Baseline appendicular lean mass, 5 adjustments, and composite outcomes for mortality or disability: Men (*n* = 1026)

					Crude				Age-adjusted				Multivariate-adjusted^a^				
	Mean (SD)	*n*	Mortality or disability, *n* (%)	*P* ^*^	HR	(95% CI)	*P*	*P for trend*	HR	(95% CI)	*P*	*P for trend*	HR	(95% CI)	*P*	*P for trend*	AUC^b^
ALM, kg																	
Q1	16.33 (1.16)	205	78 (38.0)	<.001	Reference				Reference				Reference				
Q2	18.58 (0.44)	205	51 (24.9)		0.59	(0.41–0.83)	.003	.004	0.99	(0.69–1.41)	.952	.307	0.96	(0.64–1.41)	.821	.770	.855
Q3	20.08 (0.40)	205	47 (22.9)		0.52	(0.36–0.75)	.001		1.14	(0.78–1.64)	.479		1.06	(0.69–1.63)	.780		
Q4	21.50 (0.44)	205	37 (18.0)		0.41	(0.27–0.60)	<.001		1.04	(0.69–1.53)	.856		0.88	(0.55–1.41)	.611		
Q5	24.04 (1.58)	206	24 (11.7)		0.25	(0.16–0.40)	<.001		1.49	(0.89–2.40)	.115		1.15	(0.60–2.17)	.662		
ALM/leg length, kg/m																	
Q1	20.83 (1.27)	205	74 (36.1)	<.001	Reference				Reference				Reference				
Q2	23.34 (0.55)	205	52 (25.4)		0.63	(0.45–0.91)	.014	.006	1.04	(0.73–1.50)	.821	.152	0.97	(0.65–1.44)	.967	.549	.859
Q3	24.99 (0.44)	205	54 (26.3)		0.66	(0.47–0.94)	.023		1.24	(0.85–1.73)	.281		1.07	(0.70–1.64)	.107		
Q4	26.53 (0.50)	205	26 (12.7)		0.29	(0.18–0.45)	<.001		0.73	(0.46–1.14)	.178		0.57	(0.32–1.00)	.057		
Q5	29.34 (1.69)	206	31 (15.0)		0.36	(0.23–0.54)	<.001		1.70	(1.07–2.64)	.021		1.25	(0.66–2.34)	.125		
ALM/height, kg/m																	
Q1	10.21 (0.62)	205	73 (35.6)	<.001	Reference				Reference				Reference				
Q2	11.41 (0.26)	205	51 (24.9)		0.64	(0.44–0.91)	.014	.017	1.02	(0.70–1.45)	.108	.572	0.97	(0.65–1.46)	.892	.720	.858
Q3	12.19 (0.21)	205	51 (24.9)		0.63	(0.44–0.89)	.010		1.26	(0.87–1.80)	.251		1.18	(0.76–1.82)	.459		
Q4	12.91 (0.22)	205	31 (15.1)		0.36	(0.24–0.55)	<.001		0.78	(0.50–1.18)	.214		0.64	(0.38–1.07)	.009		
Q5	14.20 (0.74)	206	31 (15.0)		0.36	(0.25–0.55)	<.001		1.43	(0.91–2.21)	.094		1.07	(0.56–2.03)	.829		
ALM/(height)^2^, kg/m^2^																	
Q1	6.29 (0.35)	205	62 (30.2)	.025	Reference				Reference				Reference				
Q2	6.96 (0.13)	205	51 (24.9)		0.79	(0.54–1.14)	.206	.184	1.07	(0.73–1.55)	.733	.484	1.09	(0.71–1.65)	.703	.910	.854
Q3	7.39 (0.12)	205	46 (22.4)		0.69	(0.49–1.02)	.061		1.18	(0.80–1.74)	.391		1.10	(0.69–1.74)	.704		
Q4	7.81 (0.14)	205	43 (21.0)		0.64	(0.43–0.95)	.027		1.01	(0.68–1.49)	.956		0.87	(0.52–1.45)	.595		
Q5	8.50 (0.38)	206	35 (17.0)		0.52	(0.34–0.78)	.002		1.39	(0.90–2.11)	.132		1.13	(0.58–2.18)	.722		
ALM/weight, kg/kg × 100																	
Q1	29.22 (1.00)	205	74 (36.1)	<.001	Reference				Reference				Reference				
Q2	31.13 (0.36)	205	41 (20.0)		0.51	(0.35–0.75)	.001	.110	0.86	(0.58–1.25)	.431	.580	0.94	(0.62–1.39)	.741	.735	.854
Q3	32.30 (0.30)	205	46 (22.4)		0.58	(0.40–0.83)	.004		0.91	(0.62–1.31)	.603		0.99	(0.67–1.44)	.950		
Q4	33.49 (0.38)	205	42 (20.5)		0.52	(0.35–0.75)	.001		0.85	(0.58–1.24)	.404		0.95	(0.62–1.44)	.812		
Q5	35.59 (1.20)	206	34 (16.5)		0.40	(0.26–0.59)	<.001		0.80	(0.52–1.20)	.286		0.91	(0.55–1.47)	.703		
ALM/BMI, kg/kg/m^2^ × 10																	
Q1	7.51 (0.37)	205	80 (39.0)	<.001	Reference				Reference				Reference				
Q2	8.26 (0.16)	205	60 (29.3)		0.70	(0.50–0.98)	.037	.002	0.97	(0.69–1.36)	.877	.762	1.02	(0.72–1.43)	.904	.930	.854
Q3	8.74 (0.13)	205	45 (22.0)		0.48	(0.33–0.70)	<.001		0.94	(0.65–1.36)	.756		1.03	(0.70–1.50)	.891		
Q4	9.29 (0.16)	205	32 (15.6)		0.33	(0.22–0.49)	<.001		0.93	(0.60–1.41)	.737		0.94	(0.60–1.50)	.795		
Q5	10.19 (0.52)	206	20 (9.7)		0.21	(0.12–0.33)	<.001		0.87	(0.51–1.42)	.589		1.05	(0.60–1.80)	.855		
AWGS criteria^c^																	
No sarcopenia	–	1015	228 (22.5)	<.001	Reference				Reference				Reference				
Sarcopenia		11	9 (81.8)		6.02	(2.86–11.04)	<.001		2.12	(1.00–3.92)	.029		2.27	(1.04–4.41)	.024	–	–

*ALM* appendicular lean mass, *HR* hazard ratio, *CI* confidence interval, *SD* standard deviation, *AUC* area under the curve, *BMI* body mass index, *AWGS* Asian Working Group for Sarcopenia

^*^The χ^2^ test

^a^Adjusted for age, smoking status, education, family income, and history of stroke, hypertension, heart disease, hyperlipidemia, and diabetes mellitus

^b^The AUC was calculated with the multivariate-adjusted logistic regression model

^c^AWGS defined sarcopenia in elderly men as low skeletal muscle mass (<7.0 kg/m^2^ using dual X-ray absorptiometry) plus low muscle strength (handgrip strength <26 kg) and/or low physical performance (usual gait speed <0.8 m/s)

**Table 3:** Baseline appendicular lean mass, 5 adjustments, and composite outcomes for mortality or disability: Women (n = 952)

					Crude				Age-adjusted				Multivariate-adjusted^a^				
	Mean (SD)	*n*	Mortality or disability, *n* (%)	*P* ^*^	HR	(95% CI)	*P*	*P for trend*	HR	(95% CI)	*P*	*P for trend*	HR	(95% CI)	*P*	*P for trend*	AUC^b^
ALM, kg																	
Q1	11.59 (0.72)	190	49 (25.8)	<.001	Reference				Reference				Reference				
Q2	12.93 (0.25)	190	32 (16.8)		0.61	(0.39–0.95)	.031	.050	0.88	(0.56–1.37)	.582	.659	0.86	(0.54–1.35)	.508	.669	.807
Q3	13.94 (0.30)	191	25 (13.1)		0.46	(0.28–0.74)	.002		0.84	(0.51–1.36)	.487		0.74	(0.44–1.25)	.268		
Q4	14.99 (0.30)	190	33 (17.4)		0.61	(0.39–0.95)	.029		1.20	(0.76–1.88)	.434		1.15	(0.69–1.91)	.581		
Q5	16.93 (1.33)	191	13 (6.8)		0.24	(0.12–0.42)	<.001		0.72	(0.37–1.33)	.316		0.64	(0.30–1.28)	.222		
ALM/leg length, kg/m																	
Q1	15.95 (0.89)	190	44 (23.2)	.009	Reference				Reference				Reference				
Q2	17.65 (0.35)	190	30 (15.8)		0.63	(0.39–1.00)	.053	.106	0.88	(0.55–1.39)	.583	.937	0.81	(0.49–1.31)	.392	.964	.803
Q3	18.79 (0.34)	191	29 (15.2)		0.60	(0.37–0.96)	.033		0.88	(0.53–1.40)	.590		0.76	(0.44–1.29)	.310		
Q4	19.98 (0.37)	190	31 (16.3)		0.64	(0.40–1.00)	.056		1.14	(0.73–1.80)	.593		1.03	(0.59–1.80)	.917		
Q5	22.35 (1.66)	191	18 (9.4)		0.37	(0.21–0.63)	<.001		0.90	(0.51–1.56)	.714		0.77	(0.37–1.57)	.482		
ALM/height, kg/m																	
Q1	7.84 (0.45)	190	38 (20.0)	.141	Reference				Reference				Reference				
Q2	8.63 (0.16)	190	31 (16.3)		0.77	(0.48–1.24)	.290	.150	0.95	(0.59–1.53)	.998	.722	0.90	(0.54–1.48)	.840	.714	.803
Q3	9.19 (0.17)	191	30 (15.7)		0.75	(0.46–1.20)	.227		0.90	(0.55–1.45)	.393		0.83	(0.49–1.40)	.499		
Q4	9.78 (0.17)	190	33 (17.4)		0.81	(0.51–1.30)	.387		1.23	(0.76–1.96)	.669		1.21	(0.69–2.11)	.481		
Q5	10.87 (0.79)	191	20 (10.5)		0.49	(0.28–0.84)	.010		1.00	(0.57–1.72)	.833		0.93	(0.45–1.48)	.675		
ALM/(height)^2^, kg/m^2^																	
Q1	5.21 (0.28)	190	25 (13.2)	.744	Reference				Reference				Reference				
Q2	5.72 (0.10)	190	33 (17.4)		1.33	(0.79–2.26)	.283	.695	1.61	(0.96–2.73)	.074	.927	1.56	(0.91–2.69)	.110	.831	.804
Q3	6.06 (0.10)	191	34 (17.8)		1.35	(0.81–2.29)	.250		1.44	(0.86–2.43)	.170		1.47	(0.84–2.62)	.186		
Q4	6.42 (0.10)	190	29 (15.3)		1.15	(0.67–1.98)	.611		1.46	(0.86–2.52)	.166		1.54	(0.82–2.93)	.183		
Q5	7.11 (0.47)	191	31 (16.2)		1.23	(0.73–2.25)	.444		1.40	(0.83–2.40)	.207		1.56	(0.77–3.16)	.216		
ALM/weight, kg/kg × 100																	
Q1	23.88 (0.97)	190	34 (17.9)	.607	Reference				Reference				Reference				
Q2	25.64 (0.36)	190	29 (15.3)		0.84	(0.53–1.34)	.503	.485	0.95	(0.58–1.56)	.849	.402	1.02	(0.61–1.72)	.933	.249	.802
Q3	26.80 (0.34)	191	25 (13.1)		0.72	(0.43–1.21)	.217		0.88	(0.52–1.46)	.619		0.98	(0.55–1.73)	.947		
Q4	27.97 (0.34)	190	35 (18.4)		1.07	(0.67–1.72)	.781		1.19	(0.74–1.91)	.472		1.43	(0.83–2.47)	.196		
Q5	30.15 (1.32)	191	29 (15.2)		0.87	(0.53–1.43)	.594		1.10	(0.67–1.81)	.700		1.36	(0.75–2.45)	.311		
ALM/BMI, kg/kg/m^2^ × 10																	
Q1	5.17 (0.32)	190	45 (23.7)	<.001	Reference				Reference				Reference				
Q2	5.79 (0.12)	190	48 (25.3)		1.12	(0.75–1.69)	.578	.042	1.53	(1.02–2.31)	.041	.822	1.63	(1.06–2.49)	.019	.986	.808
Q3	6.16 (0.11)	191	30 (15.7)		0.64	(0.40–1.01)	.059		1.07	(0.67–1.70)	.770		1.16	(0.70–1.88)	.635		
Q4	6.57 (0.13)	190	13 (6.8)		0.27	(0.14–0.49)	<.001		0.61	(0.31–1.10)	.118		0.62	(0.31–1.19)	.227		
Q5	7.34 (0.44)	191	16 (8.4)		0.34	(0.18–0.59)	<.001		1.53	(0.54–1.77)	.997		1.15	(0.59–2.16)	.716		
AWGS criteria^c^																	
No sarcopenia	–	930	147 (15.8)	.381	Reference				Reference				Reference				
Sarcopenia		22	5 (22.7)		1.48	(0.53–3.25)	.388		0.76	(0.27–1.67)	.545		0.78	(0.27–1.75)	.588		–

*ALM* appendicular lean mass, *HR* hazard ratio, *CI* confidence interval, *SD* standard deviation, *AUC* area under the curve, *BMI* body mass index, *AWGS* Asian Working Group for Sarcopenia

^*^The χ^2^ test

^a^Adjusted for age, smoking status, education, family income, and history of stroke, hypertension, heart disease, hyperlipidemia, and diabetes mellitus

^b^The AUC was calculated with the multivariate-adjusted logistic regression model

^c^AWGS defined sarcopenia in elderly women as low skeletal muscle mass (<5.4 kg/m^2^ using dual X-ray absorptiometry) plus low muscle strength (handgrip strength <18 kg) and/or low physical performance (usual gait speed <0.8 m/s)

The coincidence rate of the lowest quintile among each ALM index (ALM and adjusted ALMs) are shown in Additional file 1: Table S1. In men and women, there were higher coincident rates (more than 80%) between ALM and ALM/leg length, ALM/height indexes, or ALM/leg length and ALM/height indexes, or ALM/height and ALM/height^2^ indexes, respectively.

We divided the cohort into quintiles and determined HRs in crude, age-adjusted and multivariate-adjusted models. Men with higher ALM, ALM/leg length, ALM/height, and ALM/BMI values displayed a lower risk for mortality or disability (crude), although these relationships disappeared after adjusting for age. In the multivariate-adjusted model, none of the ALM indices was positively associated with outcomes. Women with high ALM and higher ALM/BMI values displayed a lower risk for mortality or disability (crude), although these relationships again disappeared after adjusting for age. In the multivariate-adjusted model, none of the ALM indices was positively associated with outcomes. The AUC was the highest in the ALM/leg length followed by ALM/height in men. In women, the AUC was the highest in the ALM/BMI followed by ALM.

Additional file 2: Table S2 shows mixed-effects analyses of the fixed effects of ALM indices over 12 years. The fixed effect of age and the interaction of age and time on ALM and the 5 ALM adjustments were statistically significant in men. In women, both the fixed effect of age and the interaction of age and time were statistically significant for ALM, ALM/leg length, ALM/height, and ALM/weight. Slopes of the ALM and 5 ALM adjustments in men and of ALM, ALM/leg length, ALM/height, and ALM/weight in women thus differed by age. The interaction of age and time was negative (β < 0, *P* < .001) for each ALM index in men and for ALM, ALM/leg length, and ALM/height in women, and the interaction was positive (β > 0, *P* < .05) only for ALM/weight in women. Therefore, the slope of each ALM index in men and ALM, ALM/leg length, and ALM/height in women differed by age at baseline. When we adjusted lifestyle-related factors including smoking status, alcohol intake, total physical activity, and energy intake, the association between age and muscle mass decline remained positive (data not shown). This finding means the indexes of decline in age-related muscle mass were independent of these lifestyle-related factors.

When we estimated the slope of ALM and the 5 ALM adjustments according to age at baseline, each ALM index among men started to decrease at 49 years old for ALM (49-year slope, −0.012 kg/year, *P* = .013, Additional file 3: Figure S1A), at 60 years old for ALM/leg length (60-year slope, −0.017 kg/m/year, *P* = .003, Additional file 3: Figure S1B), at 50 years old for ALM/height (50-year slope, −0.006 kg/m/year, *P* = .033, Additional file 3: Figure S1C), at 52 years old for ALM/height^2^ (52-year slope, −0.004 kg/m^2^/year, *P* = .017, Additional file 3: Figure S1D), at 50 years old for ALM/weight (50-year slope, −0.013 kg/kg × 100/year, *P* = .038, Additional file 3: Figure S1E), and at 45 years old for ALM/BMI (45-year slope, −0.005 kg/kg/m^2^ × 10/year, *P* = .025, Additional file 3: Figure S1F).

For women, 3 ALM indices—ALM, ALM/leg length, and ALM/height—started to decrease at 40 years old for ALM (40-year slope, −0.019 kg/year, *P* < .001, Additional file 3: Figure S1A), at 45 years old for ALM/leg length (45-year slope, −0.014 kg/m/year, *P* = .029, Additional file 3: Figure S1B), and at 40 years old for ALM/height (40-year slope, −0.011 kg/m/year, *P* = .002, Additional file 3: Figure S1C).

In the results summary, Crude Cox proportional hazard models and AUC indicated that higher ALM and ALM/BMI in women, and higher ALM, ALM/leg length, ALM/height, and ALM/BMI in men were associated with lower risks for mortality or disability than ALM/height^2^. The higher coincidence rates of the lowest quintile among each ALM index were shown between ALM and ALM/leg length, ALM/height indexes, or ALM/leg length and ALM/height indexes, or ALM/height and ALM/height^2^ indexes in men and women, respectively. In addition, additional analyses using the mixed effect model indicated all ALM indexes in men, and ALM, ALM/leg length, and ALM/height in women could better predict age-related lean muscle mass decline. Considering the age-related muscle mass decline, unadjusted ALM would be the first variable to assess, regardless of sex, in this Japanese cohort study.

## Discussion

This study indicated that crude ALM and ALM/BMI values in women and crude ALM, ALM/leg length, ALM/height, and ALM/BMI in men were positively associated with lower risks for mortality or disability, respectively. Data in the mixed effect model showed that interactions of age and time were negative for each ALM index in men (β for age × time < 0, *P* < .001) and for ALM, ALM/leg length, and ALM/height in women (β for age × time < 0, *P* < .05). The decreasing trend in these indices was greater among the elderly. Considering all results, including those in the mixed effect model, crude ALM for both sexes and ALM/leg length or ALM/height for men only appear more appropriate for predicting future mortality or disability compared with ALM/height^2^, which is currently used in the AWGS definition. Thus no adjustment for ALM to predict mortality or disability, regardless of sex, would be the best assessment, as it could reflect age-related muscle mass decline in both sexes.

Previous cross-sectional studies among Japanese subjects have indicated that the percentage of total skeletal muscle mass index with height adjustment decreased by 10.8% in men and by 6.4% in women among those aged 40 to 79 years [21], and that lean body mass divided by height^2^ and appendicular muscle mass divided by height^2^ were associated with grip strength [22]. Cross-sectional analysis showed that skeletal muscle mass index decreased with age only in men in the same Japanese cohort as this study [10]. In contrast, for women, SMI with height-adjustment (ALM/height^2^) was shown to represent a poor predictor of muscle mass decline among Chinese [13], Korean [9], and Japanese subjects [10]. The major reasons for sex differences in previous reports have been considered to be based on differences in body mass, body fat mass, hormones, and daily physical activity between men and women.

In terms of body fat, some studies have indicated that age-related fat mass [23, 24], height and fat mass, and BMI are all better for predicting disability [25, 26] or the prevalence of sarcopenia [27] than the ALM/height^2^ method [28]. In the present study, higher ALM/BMI was associated with lower risks for mortality or disability in both sexes, but the effect of the interaction of age and time on ALM/BMI was not significant in women. This means ALM/BMI does not decline with age in women; in other words, the index did not work well to predict age-related muscle mass decline in our cohort study.

In addition, ALM/weight did not predict future mortality or disability and did not show any decreasing trend with age. Thinness in young women represents a serious health concern in Japan [29], and age differences in weight or BMI may be seriously affected by cohort differences. In sub-analyses, we examined associations between ALM-adjusted fat mass (%) as measured by DXA, and mortality or disability, but found no significant associations between these variables in women (data not shown). However, men with a higher ALM/body fat showed lower risks for mortality or disability (hazard ratio [95% confidence interval], Q1; reference, Q2; 0.80 [0.57–1.13], Q3; 0.57 [0.39–0.83], Q4; 0.43 [0.28–0.64], Q4; 0.36 [0.23–0.54], *P* for trend <.001). Because body mass and body fat are generally lower in Japanese subjects than in Caucasians [30], adjusting for fat mass may be less useful when evaluating the age-related muscle mass decline among relatively lean ethnic groups.

In this study, we first considered leg length for ALM adjustment. One reason for the poor association between age and ALM/height^2^ among women was thought to be that adjustment using height^2^ may result in over-adjustment for the elderly. We have previously reported a longitudinal decreasing trend in height according to baseline age in the same cohort used in this study (age, 40–79 years) [31]. Loss of height began in men at 41 years and in women at 42 years, and the slope of height was −0.01 to −0.17 cm/year among men 41–79 years and −0.02 to −0.25 cm/year among women 42–79 years [31]. This means that the age-related decreasing trend in height was greater in women than in men, and that height^2^ adjustment may result in an over-adjustment for women.

Several limitations must be considered when interpreting the results of this study. First, we assessed ALM using DXA measurements. Although DXA is one of the best ways to assess muscle mass, infiltration of fat into the muscle is difficult to distinguish [10, 32]. We compared the ALM adjustment of weight or BMI in this study, and these variables might be a better predictor of fat mass than DXA measurements. Reproducibility using different modalities such as computed tomography or bioelectrical impedance needs to be examined in future studies. Second, age- or multivariate-adjusted hazard ratios of ALM indices for disability or mortality were not statistically significant, but age should be a strong predictor of these outcomes. Additional analyses using the mixed effect model indicated all ALM indexes in men, and ALM, ALM/leg length, and ALM/height in women could better predict age-related lean mass decline. When we adjusted for confounding lifestyle-related factors, including smoking status, alcohol intake, total physical activity, and energy intake, the association for age-related muscle mass decline remained; therefore, non-adjusted ALM index could better predict age-related muscle mass decline both in men and women.

The main strengths of the present study are as follows: 1) the longitudinal design of our analyses lends strength to our inferences, as each individual was followed for more than 15 years, providing evidence of a causal association between ALM indices and disability; 2) use of a middle- and older-aged sample of randomly selected age- and sex-stratified non-institutionalized individuals from the community means that our results may be applicable to non-institutionalized Japanese elderly individuals; and 3) this is the first study to assess multiple ALM indices concurrently.

## Conclusions

Longitudinal data showed that crude ALM for both sexes and ALM/leg length, ALM/height, or ALM/BMI for men are more appropriate to predict future disability compared to ALM/height^2^, which is currently used in the AWGS. Considering the age-related muscle mass decline, unadjusted ALM would be the first variable to assess, regardless of sex, in this Japanese cohort study.

Further studies are needed to support reproducibility of these results concerning the most appropriate methods for measuring muscle mass in Asian women.

## Additional files

Additional file 1: Table S1. Coincidence rate of the lowest quintile among each variable (XLSX 10 kb)

Additional file 2: Table S2. Mixed-effects analyses of fixed effects for ALM and 5 adjustments of ALM over 12 years* (XLSX 27 kb)

Additional file 3: Figure S1. A. Estimated linear changes in ALM over 12 years by 4-year age groups at baseline. B. Estimated linear changes in ALM/leg length over 12 years by 4-year age groups at baseline. C. Estimated linear changes in ALM/height over 12 years by 4-year age groups at baseline. D. Estimated linear changes in ALM/height^2^ over 12 years by 4-year age groups at baseline. E. Estimated linear changes in ALM/weight over 12 years by 4-year age groups at baseline. F. Estimated linear changes in ALM/BMI over 12 years by 4-year age groups at baseline. (XLSX 181 kb)
